# Occupational benzene exposure and risk of kidney and bladder cancers: a systematic review and meta-analysis

**DOI:** 10.1097/CEJ.0000000000000911

**Published:** 2024-08-20

**Authors:** Monireh Sadat Seyyedsalehi, Mattia Bonetti, Darshi Shah, Vincent DeStefano, Paolo Boffetta

**Affiliations:** a Department of Medical and Surgical Sciences, University of Bologna, Bologna, Italy; b Department of Family, Population and Preventive Medicine, Renaissance School of Medicine; c Stony Brook Cancer Center, Stony Brook University, Stony Brook, New York, USA

**Keywords:** benzene, bladder cancer, kidney cancer, occupational exposure

## Abstract

**Introduction:**

Benzene is recognized as leukemogenic. However, the association between it and solid cancers has been the subject of less investigation. We aim to conduct a systematic review and meta-analysis to evaluate the association between occupational exposure to benzene and the risk of urinary tract cancer, including kidney and bladder.

**Methods:**

We included 41 cohort and case–control studies listed in the most recent International Agency for Research on Cancer (IARC) Monograph on benzene exposure and the result of a literature review to identify more recent studies. Forest plots of relative risk (RR) were constructed for kidney, bladder, and urinary tract cancer overall. A random-effects model was used to address heterogeneity between studies. Stratified analyses were conducted to explore effect modification.

**Results:**

Our findings revealed an association between exposure to occupational benzene and kidney and unspecified urinary tract cancers (RR = 1.20, 95% confidence interval = 1.03–1.39), and an association of borderline statistical significance with bladder cancer (RR = 1.07, 95% confidence interval = 0.97–1.18). Publication bias was excluded for both kidney (*P* = 0.809) and bladder cancer (*P* = 0.748). Stratification analysis according to the selected study characteristics showed no difference except regarding the industry for kidney cancer (*P* < 0.000), with a stronger association in the chemical industry. An analysis by exposure level did not reveal any trend for kidney cancer, whereas there was a trend (*P* = 0.01) for bladder cancer.

**Conclusion:**

Our study found an association between occupational benzene exposure and kidney cancer and a dose-effect association between benzene exposure and bladder cancer.

## Introduction

Benzene is an organic, aromatic hydrocarbon that has been used in a variety of industries (e.g. plastics, dyes, lubricants, synthetic fibers) as a solvent and reagent (Galbraith *et al*., 2010). Nowadays, it is most frequently found as a contaminant, for example, in gasoline. Benzene is highly toxic and prolonged exposure to it can lead to serious health effects. Benzene has long been recognized as leukemogenic and is classified as a group 1 carcinogen by the International Agency for Research on Cancer (IARC) (IARC, 2018). There has been some question as to whether benzene exposure increases the risk of solid cancers, which could represent a significant contribution to the burden of cancer in addition to its impact as a leukemogen; but despite several studies have reported results on the topic, evidence is inconclusive (Sassano *et al.*, 2024; Cox, 1991; Falzone *et al*., 2016; Loomis *et al*., 2017) and systematic reviews are lacking.

Among different types of tumors, urinary cancers including kidney (age-standardized rate: 4.6 per 100 000) and bladder and other urinary tract (age-standardized rate: 5.6 per 100 000) cancer accounts for about 5.5% of cancers worldwide, with higher incidence in high-income countries (Sung *et al*., 2021). Urinary cancers occur more frequently in men than in women, with a ratio of 2.5 : 1 globally (Sung *et al*., 2021). Numerous factors have been implicated in the development of urinary organ cancer, including occupational, lifestyle, and genetic factors. Among them, smoking, workplace exposures, overweight, lack of physical activity, poorly controlled hypertension, alcohol consumption, diet, and schistosoma infection are known risk factors. This makes urinary organ cancers an excellent candidate for prevention strategies (Scélo, 2007; Chow *et al*., 2010; Padala *et al*., 2020; Saginala *et al*., 2020). In previous studies, aromatic amines, such as benzidine, 2-naphthylamine, and 4-aminobiphenyl, as well as solvents, polycyclic aromatic hydrocarbons (PAHs), and some heavy metals, were found to be occupational risk factors for kidney and bladder cancer. Until now, however, no systematic review or meta-analysis has investigated the association between benzene exposure and these two cancer types (Pesch *et al*., 2000a; Letašiová *et al*., 2012).

We aim to conduct a systematic review and meta-analysis of occupational cohort and case–control studies, to evaluate the association between exposure to benzene and kidney, bladder, and urinary tract cancer incidence and mortality by considering some available related factors such as sex, country, and so on.

## Methods

### Data sources, search strategy, selection criteria and quality assessment

Our study protocol was registered in the PROSPERO database (Registration No. CRD42022379720); and we followed the COSMOS-E and PRISMA-statement to conduct and report this systematic review and meta-analysis (Supplementary Table 1, Supplemental digital content 1, http://links.lww.com/EJCP/A478) (Moher *et al*., 2009).

**Fig. 1:**
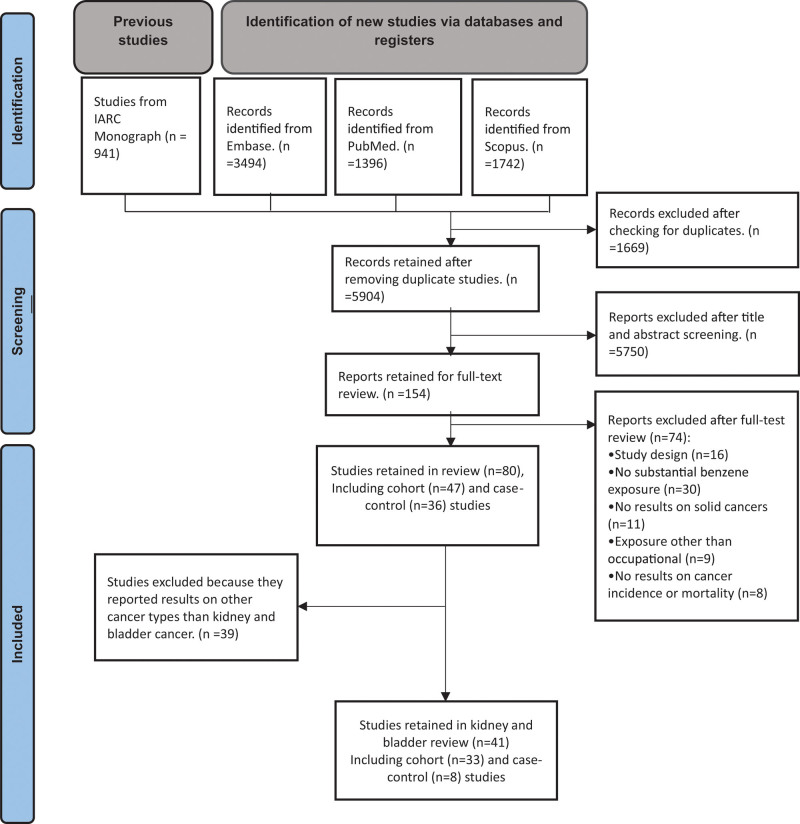
Selection of studies for inclusion in the review and meta-analysis. IARC, International Agency for Research on Cancer.

Figure 1 shows the flow diagram of the literature search and study selection process. First, we included all studies that were reported in the most recent IARC Monograph on benzene exposure published in 2018 (IARC, 2018). Next, we conducted a search in the *MEDLINE* (*PubMed*), *SCOPUS*, and *EMBASE* (*Ovid*) databases for studies reported after that publication. Two authors (M.S.S. and M.B.) performed the search independently. The final search was updated in May 2024 for English-, French-, Italian-, German-, and Spanish-language cohort and case–control peer-reviewed publications published on the association of occupational exposure to benzene and risk (incidence and mortality) of any type of solid cancer.

The search strategy was designed using MeSH terms like ((‘neoplasms’[Title/Abstract] OR ‘carcinoma’[Title/Abstract] OR ‘cancer’[Title/Abstract] OR ‘malignant’[Title/Abstract]) AND (‘benzene’[All Fields] OR ‘benzol’[All Fields] OR (‘cyclohexa-1’[All Fields] OR ‘3 5 triene’[All Fields]) OR ‘5-cyclohexatriene’[All Fields]) OR ‘cyclohexatriene’[All Fields]). The complete search string is reported in Supplementary Table 2, Supplemental digital content 1, http://links.lww.com/EJCP/A478.

Four authors (M.S.S., M.B., D.S., and V.D.) independently reviewed the list of titles, abstracts, and full text of the paper. If multiple reports were based on the same database, we included only the most informative report, typically based on the most recent update. We included cohort studies, including nested case–control analyses, of workers employed in industries and occupations in which benzene represents a major source of exposure. These include the petroleum industry (all phases: extraction, refining, distribution, gas station), shoemakers, paint production and painters, chemical industry (to be checked), rubber industry (to be checked), printing, and laboratory workers. We excluded studies of workers mainly exposed to other carcinogens such as PAH (including diesel exhaust, coke oven, aluminum production, and firefighting), silica, and butadiene. We included community-based case–control studies if they reported results on exposure to benzene assessed via a job-exposure matrix or expert evaluation; results of such studies based on employment in specific industries and occupations were excluded because they were mainly based on small numbers. Studies involving animals, blood, tissue, genetic evaluation, and studies without full text were excluded from our review. Also, we excluded study types other than cohort and case–control designs such as letters, ecological studies, and case reports. Studies restricted to nonsolid tumors including leukemia, lymphoma, or myeloma were excluded. This strategy led to the identification of 80 independent cohort (*n* = 47) and case–control (*n* = 36) studies (Fig. 1).

The data extraction file was completed by four reviewers (M.S.S., M.B., D.S., and V.D.) independently based on the full text of potentially relevant articles. It contained the author’s name, year of publication, title, type of study, country, sample size, job title, period of employment, outcome, type of controls, type of cancer (including topography and histology), effect size measures, including relative risks (hazard risks/RRs/standardized mortality ratios) for cohort studies and odds ratios for case–control studies, and their 95% confidence interval (CI). Also, we extracted the results for subgroups (e.g. sex, different doses, and duration of exposure). If RR/odds ratio or CI were not reported, we calculated them from the row data if possible.

The quality and susceptibility to the bias of each included study were evaluated using a modified version of the Newcastle–Ottawa Scale for case–control (nine items) and cohort studies (10 items) by four independent reviewers (M.S.S., M.B., D.S., and V.D.) (Stang, 2010) (Supplementary Table 3, Supplemental digital content 1, http://links.lww.com/EJCP/A478). Studies that scored less than 8 corresponded to low quality and those that scored 8 or more were considered of high quality. During the process of all steps if there were any major discrepancies, a fifth author (P.B.) intervened.

### Examination of the association between benzene exposure and kidney and bladder cancer

For this aim, we only focused on studies with reported effect size measurements and the corresponding 95% CIs of incidence or mortality for kidney, bladder, and urinary tract cancer (ureters or unspecified urinary tract cancer) as an outcome. Because most studies report results for bladder and other urinary tract cancers together, it was difficult to run a meta-analysis for these two groups separately. All analyses were completed using the STATA, version 14.0 (StataCorp LLC, College Station, Texas, USA). We conducted a series of meta-analyses of nonoverlapping studies for kidney, bladder, and urinary tract cancer and total benzene based on a random-effects model and tested for heterogeneity among studies using the *Q* statistics and the *I*^2^ test based on the variation across studies rather than within studies (Higgins and Thompsom, 2002). In addition, we conducted stratified analyses by geographic region (Europe, North America, others including Asian, Latin America, and Australia), study design (cohort and case–control), quality score (low and high quality), outcome (incidence and mortality), control for tobacco smoking, year of publication (<2000 and ≥2000), exposure circumstance (oil industry, chemical industry, other industries, benzene exposure across multiple industries, and mixed), sex (men and women), and different dose exposure [low, medium, and high (Supplementary Table 4, Supplemental digital content 1, http://links.lww.com/EJCP/A478)]. Finally, we assessed publication bias by the visual inspection of the funnel plot and the Egger test (Egger *et al*., 1997).

## Results

**Fig. 2:**
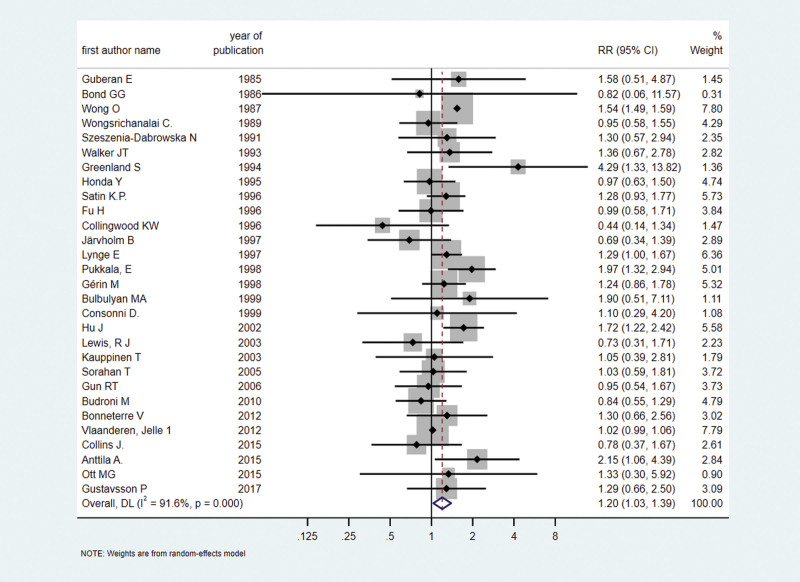
Forest plot (random-effects model) of results on the association between benzene exposure and kidney and urinary tract cancer. CI, confidence interval, RR, relative risk.

**Fig. 3:**
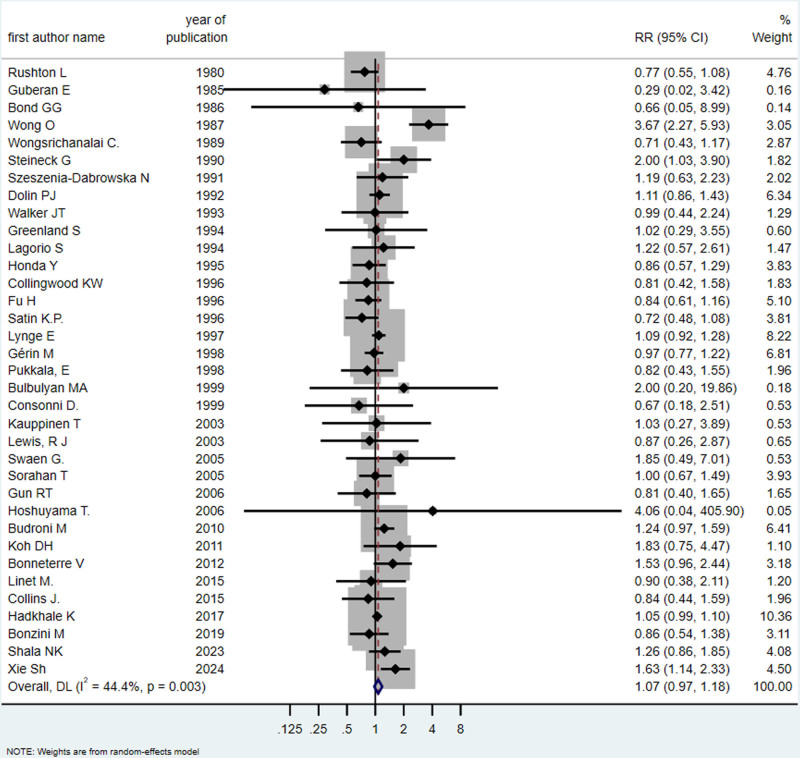
Forest plot (random-effects model) of results on the association between benzene exposure and bladder cancer. CI, confidence interval, RR, relative risk.

**Fig. 4:**
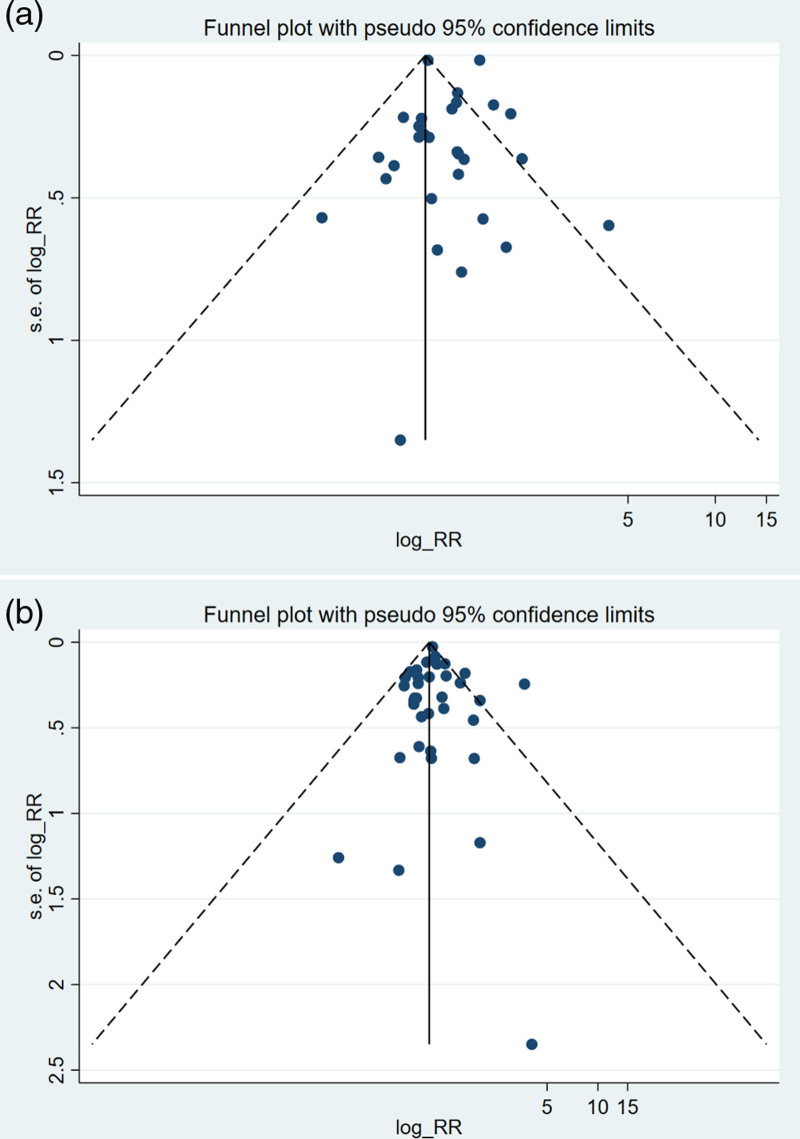
Funnel plot of results on the association between benzene exposure and kidney (a) and bladder (b) cancers. RR, relative risk.

Among the 80 studies retained in the review, 41 independent cohort (*n* = 33) and case–control (*n* =8) (Bond *et al.*, 1986; Dolin and Cook-Mozaffari, 1992; Collingwood *et al.*, 1996; Ott *et al.*, 1978; Rushton and Alderson, 1980; Guberan and Raymond, 1985; Wong, 1987; Wongsrichanalai *et al.*, 1989; Steineck *et al.*, 1990; Szeszenia-Dabrowska *et al.*, 1991; Walker *et al.*, 1993; Greenland *et al.*, 1994; Lagorio *et al.*, 1994; Honda *et al.*, 1995; Fu *et al.*, 1996; Satin *et al.*, 1996; Järvholm *et al.*, 1997; Lynge *et al.*, 1997; Gérin *et al.*, 1998; Pukkala, 1998; Bulbulyan *et al.*, 1999; Consonni *et al.*, 1999; Pesch *et al.*, 2000b; Hu *et al.*, 2002; Kauppinen *et al.*, 2003; Lewis *et al.*, 2003; Sorahan *et al.*, 2005; Swaen *et al.*, 2005; Gun *et al.*, 2006; Hoshuyama *et al.*, 2006; Budroni *et al.*, 2011; Koh *et al.*, 2011; Bonneterre *et al.*, 2012; Vlaanderen *et al.*, 2013; Anttila *et al.*, 2015; Collins *et al.*, 2015; Linet *et al.*, 2015; Gustavsson *et al.*, 2017; Hadkhale *et al.*, 2017; Shala *et al.*, 2023; Xie *et al.*, 2024) studies reported 105 different risk estimates for kidney, bladder, or urinary tract cancer, including different outcomes (men, women, both) and other stratified factors. These studies include 31 studies on kidney cancer and other urinary tract cancers and 35 studies related to bladder cancer. Details of these studies are provided in Supplementary Table 5, Supplemental digital content 1, http://links.lww.com/EJCP/A478. The findings revealed an association between exposure of occupational benzene and kidney and urinary tract cancer (RR = 1.20, 95% CI = 1.03–1.39; *I*^2^ = 91.6%, *P*_heterogeneity_ = 0.000, 31 risk estimates) (Fig. 2), and an association of borderline statistical significance with bladder cancer (RR = 1.07, 95% CI = 0.97–1.18; *I*^2^ = 44.4%, *P*_heterogeneity_ = 0.003, 35 risk estimates) (Fig. 3). Publication bias was excluded through the Egger test for kidney cancer studies (*P* = 0.809) and bladder and urinary tract cancer (*P* = 0.748); funnel plots are shown in Figure 4.

Results of stratified analyses according to selected characteristics are reported in Table 1. There was no difference between outcome (*P*_kidney_ = 0.9; *P*_bladder_ = 0.3), geographic region (*P*_kidney_ = 0.7; *P*_bladder_ = 0.9), year of publication (*P*_kidney_ = 0.2; *P*_bladder_ = 0.4), study design (*P*_bladder_ = 0.3), quality score (*P*_kidney_ = 0.2; *P*_bladder_ = 0.8), sex (*P*_kidney_ = 0.6; *P*_bladder_ = 0.7), and adjustment for tobacco smoking (*P*_kidney_ = 0.6; *P*_bladder_ = 0.1). Stratification by exposure circumstance revealed heterogeneity for kidney cancer (*P* < 0.000), with a stronger association for studies in the chemical industry (RR = 1.54, 95% CI = 1.49–1.59, five risk estimates), but not for bladder cancer (*P* = 0.3). Also, results by study design for kidney cancer show heterogeneity (*P* = 0.03) with a stronger association in case–control design (RR = 1.71, 95% CI = 1.12–2.43, four risk estimates). The analysis according to benzene exposure did not reveal any trend for either kidney (*P*_trend_ = 0.39), but we found a trend for bladder cancer (*P*_trend_ = 0.01) (Table 1). Results on the duration of benzene exposure were too sparse to allow a meta-analysis.

**Table 1:** Results of the meta-analyses by region, outcome, study design, year of publication, sex, industry, dose of exposure, adjustment for tobacco smoking, and quality score

Characteristics	Number of risk estimates	RR (95% CI)	*P*_heterogeneity_
Kidney cancer			
Region			
North America	13	1.25 (1.04–1.51)	0.7
Europe	14	1.17 (1.00–1.37)	
Others	2	1.06 (0.63–1.77)	
Study design			
Case–control	4	1.71 (1.12–2.43)	0.03
Cohort	25	1.12 (0.95–1.32)	
Quality score			
Low quality (<8)	10	1.31 (1.06–1.63)	0.2
High quality (≥8)	19	1.15 (0.97–1.36)	
Outcome			0.9
Incidence	16	1.17 (1.02–1.35)	
Mortality	17	1.18 (0.98–1.43)	
Years of publication			
<2000	18	1.28 (1.10–1.49)	0.2
≥2000	11	1.12 (0.94–1.32)	
Sex			
Men	16	1.18 (1.00–1.40)	0.6
Women	5	1.34 (1.09–1.66)	
Both sexes	16	1.23 (1.05–1.44)	
Dose category			
Low	2	1.00 (0.94–1.06)	0.2*
Medium	3	1.10 (0.89–1.35)	
High	4	1.07 (1.02–1.13)	
Industry			
Oil industry	11	1.15 (0.94–1.39)	<0.000
Chemical industry	5	1.54 (1.49–1.59)	
Other industries	6	1.38 (0.97–1.96)	
Benzene exposure across multiple industries	4	1.02 (0.99–1.05)	
Mixed	3	0.91 (0.45–1.84)	
Adjustment for tobacco smoking			
No	26	1.18 (1.01–1.39)	0.6
Yes	3	1.32 (0.90–1.92)	
Bladder cancer			
Region			
North America	12	1.06 (0.78–1.43)	0.9
Europe	18	1.06 (0.99–1.13)	
Others	5	1.08 (0.69–1.71)	
Study design			
Case–control	5	1.17 (0.95–1.45)	0.3
Cohort	30	1.04 (0.92–1.19)	
Quality score			
Low quality (<8)	14	1.07 (0.97–1.18)	0.8
High quality (≥8)	21	1.05 (0.87–1.28)	
Outcome			
Incidence	17	1.10 (1.01–1.20)	0.3
Mortality	23	0.99 (0.83–1.19)	
Years of publication			
<2000	20	1.02 (0.86–1.21)	0.4
≥2000	15	1.11 (1.01–1.21)	
Sex			
Men	22	1.07 (0.92–1.24)	0.7
Women	3	1.15 (0.94–1.40)	
Both sexes	16	1.06 (1–1.13)	
Dose category			
Low	5	1.19 (0.92–1.53)	0.01*
Medium	4	1.06 (0.99–1.13)	
High	5	1.20 (0.81–1.79)	
Industry			
Oil industry	15	0.98 (0.87–1.12)	0.3
Chemical industry	3	2.02 (0.96–4.24)	
Other industries	7	0.92 (0.71–1.19)	
Benzene exposure across multiple industries	6	1.09 (0.96–1.24)	
Mixed	4	1.08 (0.74–1.58)	
Adjustment for tobacco smoking			
No	30	1.04 (0.93–1.15)	0.1
Yes	5	1.28 (0.96–1.72)	

CI, confidence interval; RR, relative risk.

* The *P*-value of test for linear trend.

## Discussion

In this meta-analysis, we found that benzene exposure was associated with kidney cancer. Furthermore, analysis by dose–response showed a dose–effect association between occupational benzene exposure and bladder cancer.

Previous research reported that benzene exposure can cause several health effects, including nervous system disorders, digestive complications, anemia, and some chronic disease like cancer (Smith, 2010; D’Andrea and Reddy, 2018; Tongsantia, 2021). According to several reports published by IARC from 1982, 2009, and 2018, IARC classifies benzene as ‘carcinogenic to humans’, based on sufficient evidence of a causal association with acute myeloid leukemia, with positive results also for acute lymphocytic leukemia, chronic lymphocytic leukemia), multiple myeloma, and non-Hodgkin lymphoma (IARC, 2018). In addition, a few of the studies have reported that benzene exposure can increase the risk of developing certain types of solid cancers such as lung, bladder, and kidney. However, the question of benzene’s solid tumor carcinogenicity remains an open and important one to resolve (Huff, 2007; Falzone *et al*., 2016; Spatari, 2021).

People can be exposed to benzene through the inhalation of vapors in the environment and at the workplace. Dermal absorption represents an additional route of exposure. (Weisel, 2010; Falzone *et al*., 2016; Patel *et al*., 2020). After entering the body, benzene is metabolized in the liver to benzene oxide and several reactive metabolites, which might reach the bone marrow and other organs. They are excreted in urine, thus potentially affecting the urinary tract. There are two main mechanisms by which reactive benzene metabolites may induce carcinogenicity, including (1) covalent binding to DNA (genotoxicity) and (2) generation of reactive oxygen species via oxidative damage. Apart from benzene metabolites, benzene can have direct toxic effects on the body and can trigger proptosis, which can cause hematotoxicity and debilitation of the immune system (Snyder and Hedli, 1996; Rana and Verma, 2005; Guo *et al*., 2019).

Several confounding factors can affect the results of studies on kidney and bladder cancer, and it is indeed particularly important to consider them in the interpretation of results. Regarding kidney cancer, major risk factors include tobacco smoking, high body weight, hypertension, and other chronic kidney diseases (Scelo, 2018). With respect to bladder cancer, it is critical to consider tobacco smoking, overweight, diabetes, and lack of physical activity. Meanwhile, other workplace agents, such as PAH, might also act as confounders. However, most of the studies included in our review, particularly cohort studies with a focus on mortality as an outcome, did not adjust for potential nonoccupational and as well as occupational confounders. Although case–control studies might have other types of bias. The stratification analysis conducted did not indicate any heterogeneity in the results according to the characteristics we considered. However, there was a suggestion of a positive association between occupational exposure and the incidence of kidney and bladder cancer compared with mortality. This finding may be explained by timely diagnosis and treatment which could potentially help reduce the mortality rate associated with these cancers. However, this situation is possible if treatment is better for exposed workers than other patients, it is important to note that these findings are based on studies primarily conducted in developed countries where healthcare facilities and occupational safety measures are more easily accessible. Therefore, caution should be exercised when extrapolating these results to other populations or regions with different healthcare systems and working conditions. Furthermore, our meta-analysis revealed significant differences in kidney cancer risk within the industry of employment, with a higher risk in studies conducted in the chemical industry. This observation suggests that individuals working in this sector might be exposed to higher levels of benzene directly compared with other job titles, however, this result can be due to chance because of multiple comparisons.

There was no heterogeneity in the risk of kidney cancer according to an estimated level of exposure, whereas the results for high doses, but not those for low doses, suggested an association with bladder cancer risk. Some previous studies have suggested that high and longer term exposures to benzene have more association with increased cancer risk like acute myeloid leukemia (Green *et al*.,1981; Thomas *et al*., 2014). It is important to note, however, that the number of studies available for the analysis of dose–response was limited.

To our knowledge, this study is the first systematic review and meta-analysis on the association between occupational benzene exposure and cancer of the urinary tract, including kidney and bladder. We could include cohort and case–control studies and we reported results stratified for different important related factors, and for dose response. However, it is noted that there are limitations to consider. The small number of available studies on women workers, studies conducted outside North America and Europe, and studies reporting dose–response relationships can impact the interpretation and the generalizability of the findings. Another important limitation is the lack of adjustment for potential confounders, such as tobacco smoking, in most available studies, particularly those based on mortality. Furthermore, it was not possible in studies of kidney cancer to separate renal cell carcinoma from cancer of the renal pelvis. Future individual epidemiological studies should focus on sex differences and include regions that have received less attention, such as less developed countries. These studies can help identify potential variations in the associations between benzene exposure and cancer risks among different populations and provide insights into regions where protective guidelines may not be adequately addressed.

In conclusion, our study brought important insights into the association between occupational benzene exposure and kidney and bladder cancer risk. It highlights the need for recommendations and measures to minimize exposure to benzene in the workplace in industries like petroleum or chemical refineries to protect the health of workers. However, it is essential to consider the limitations, and exploring the impact of environmental sources of benzene would provide a more comprehensive understanding of the potential risks associated with this chemical. Countries and regions that have received less attention in terms of research on benzene exposure and cancer should also be included in future studies to enhance our understanding of this potential association.

## Acknowledgements

The authors thank Germana Giupponi for assistance in the identification of articles included in the review.

P.B. and M.S.S. conceived and designed the study. M.S.S., M.B., D.S, and V.D. selected the studies and extracted the data. M.S.S. and P.B. conducted the statistical analysis. M.S.S. drafted the manuscript. P.B. provided substantial comments to the manuscript.

The study was conducted with the internal resources of the participating institutions.

The data supporting this study’s findings are available from the corresponding author upon reasonable request.

### Conflicts of interest

P.B. acted as a consultant in litigation involving benzene exposure and kidney and bladder cancer, unrelated to the present work. For the remaining authors, there are no conflicts of interest.

## Supplementary Material


