# Supplementary material for: Occupational benzene exposure and risk of kidney and bladder cancers: a systematic review and meta-analysis
Source: Eur J Cancer Prev. 2024 Aug 20;34(3):205–13. doi: 10.1097/CEJ.0000000000000911 (PMC11949222; doi:10.1097/CEJ.0000000000000911)
Supplement: Supplementary file 1 [file ejcp-34-205-s001.pdf]

Supplementary Table 1a. PRISMA Checklist

Section and Topic	Item #	Checklist item	Location where item is reported
**TITLE**			
Title	1	Identify the report as a systematic review.	P1
**ABSTRACT**			
Abstract	2	See the PRISMA 2020 for Abstracts checklist.	P2
**INTRODUCTION**			
Rationale	3	Describe the rationale for the review in the context of existing knowledge.	P3
Objectives	4	Provide an explicit statement of the objective(s) or question(s) the review addresses.	P3
**METHODS**			
Eligibility criteria	5	Specify the inclusion and exclusion criteria for the review and how studies were grouped for the syntheses.	P4
Information sources	6	Specify all databases, registers, websites, organisations, reference lists and other sources searched or consulted to identify studies. Specify the date when each source was last searched or consulted.	P4
Search strategy	7	Present the full search strategies for all databases, registers and websites, including any filters and limits used.	P4
Selection process	8	Specify the methods used to decide whether a study met the inclusion criteria of the review, including how many reviewers screened each record and each report retrieved, whether they worked independently, and if applicable, details of automation tools used in the process.	P4
Data collection process	9	Specify the methods used to collect data from reports, including how many reviewers collected data from each report, whether they worked independently, any processes for obtaining or confirming data from study investigators, and if applicable, details of automation tools used in the process.	P4
Data items	10a	List and define all outcomes for which data were sought. Specify whether all results that were compatible with each outcome domain in each study were sought (e.g. for all measures, time points, analyses), and if not, the methods used to decide which results to collect.	P4-5
	10b	List and define all other variables for which data were sought (e.g. participant and intervention characteristics, funding sources). Describe any assumptions made about any missing or unclear information.	P4-5
Study risk of bias assessment	11	Specify the methods used to assess risk of bias in the included studies, including details of the tool(s) used, how many reviewers assessed each study and whether they worked independently, and if applicable, details of automation tools used in the process.	P5
Effect measures	12	Specify for each outcome the effect measure(s) (e.g. risk ratio, mean difference) used in the synthesis or presentation of results.	P5
Synthesis methods	13a	Describe the processes used to decide which studies were eligible for each synthesis (e.g. tabulating the study intervention characteristics and comparing against the planned groups for each synthesis (item #5)).	P5
	13b	Describe any methods required to prepare the data for presentation or synthesis, such as handling of missing summary statistics, or data conversions.	P5
	13c	Describe any methods used to tabulate or visually display results of individual studies and syntheses.	P5
	13d	Describe any methods used to synthesize results and provide a rationale for the choice(s). If meta-analysis was performed, describe the model(s), method(s) to identify the presence and extent of statistical heterogeneity, and software package(s) used.	P5

Section and Topic	Item #	Checklist item	Location where item is reported
	13e	Describe any methods used to explore possible causes of heterogeneity among study results (e.g. subgroup analysis, meta-regression).	P5
	13f	Describe any sensitivity analyses conducted to assess robustness of the synthesized results.	P5
Reporting bias assessment	14	Describe any methods used to assess risk of bias due to missing results in a synthesis (arising from reporting biases).	P5
Certainty assessment	15	Describe any methods used to assess certainty (or confidence) in the body of evidence for an outcome.	P5
**RESULTS**			
Study selection	16a	Describe the results of the search and selection process, from the number of records identified in the search to the number of studies included in the review, ideally using a flow diagram.	P6,13
	16b	Cite studies that might appear to meet the inclusion criteria, but which were excluded, and explain why they were excluded.	P13
Study characteristics	17	Cite each included study and present its characteristics.	P6
Risk of bias in studies	18	Present assessments of risk of bias for each included study.	P6
Results of individual studies	19	For all outcomes, present, for each study: (a) summary statistics for each group (where appropriate) and (b) an effect estimates and its precision (e.g. confidence/credible interval), ideally using structured tables or plots.	P6
Results of syntheses	20a	For each synthesis, briefly summarise the characteristics and risk of bias among contributing studies.	P6
	20b	Present results of all statistical syntheses conducted. If meta-analysis was done, present for each the summary estimate and its precision (e.g. confidence/credible interval) and measures of statistical heterogeneity. If comparing groups, describe the direction of the effect.	P6
	20c	Present results of all investigations of possible causes of heterogeneity among study results.	P6
	20d	Present results of all sensitivity analyses conducted to assess the robustness of the synthesized results.	P6
Reporting biases	21	Present assessments of risk of bias due to missing results (arising from reporting biases) for each synthesis assessed.	P6
Certainty of evidence	22	Present assessments of certainty (or confidence) in the body of evidence for each outcome assessed.	P6
**DISCUSSION**			
Discussion	23a	Provide a general interpretation of the results in the context of other evidence.	P7
	23b	Discuss any limitations of the evidence included in the review.	P8
	23c	Discuss any limitations of the review processes used.	P8
	23d	Discuss implications of the results for practice, policy, and future research.	P9
**OTHER INFORMATION**			
Registration and protocol	24a	Provide registration information for the review, including register name and registration number, or state that the review was not registered.	P4
	24b	Indicate where the review protocol can be accessed, or state that a protocol was not prepared.	NA
	24c	Describe and explain any amendments to information provided at registration or in the protocol.	NA
Support	25	Describe sources of financial or non-financial support for the review, and the role of the funders or sponsors in the review.	P9

Section and Topic	Item #	Checklist item	Location where item is reported
Competing interests	26	Declare any competing interests of review authors.	P9
Availability of data, code and other materials	27	Report which of the following are publicly available and where they can be found: template data collection forms; data extracted from included studies; data used for all analyses; analytic code; any other materials used in the review.	NA

Supplementary Table 1b. PRISMA Abstract Checklist

Section and Topic	Item #	Checklist item	Reported (Yes/No)
**TITLE**			
Title	1	Identify the report as a systematic review.	Yes
**BACKGROUND**			
Objectives	2	Provide an explicit statement of the main objective(s) or question(s) the review addresses.	Yes
**METHODS**			
Eligibility criteria	3	Specify the inclusion and exclusion criteria for the review.	Yes
Information sources	4	Specify the information sources (e.g. databases, registers) used to identify studies and the date when each was last searched.	Yes
Risk of bias	5	Specify the methods used to assess risk of bias in the included studies.	Yes
Synthesis of results	6	Specify the methods used to present and synthesise results.	Yes
**RESULTS**			
Included studies	7	Give the total number of included studies and participants and summarise relevant characteristics of studies.	Yes
Synthesis of results	8	Present results for main outcomes, preferably indicating the number of included studies and participants for each. If meta-analysis was done, report the summary estimate and confidence/credible interval. If comparing groups, indicate the direction of the effect (i.e. which group is favoured).	Yes
**DISCUSSION**			
Limitations of evidence	9	Provide a brief summary of the limitations of the evidence included in the review (e.g. study risk of bias, inconsistency and imprecision).	Yes
Interpretation	10	Provide a general interpretation of the results and important implications.	Yes
**OTHER**			
Funding	11	Specify the primary source of funding for the review.	Yes
Registration	12	Provide the register name and registration number.	Yes

Supplementary Table 2. Detailed search strategy used on the different databases.

**Database**	**Search string**
PubMed	(("neoplasms"[Title/Abstract] OR "carcinoma"[Title/Abstract] OR "cancer"[Title/Abstract] OR "malignant"[Title/Abstract]) AND ("benzene"[Title/Abstract] OR "benzol"[Title/Abstract] OR ("cyclohexa-1"[All Fields] AND "3 5 triene"[Title/Abstract]) OR (("1"[All Fields] AND "3"[All Fields]) AND "5-cyclohexatriene"[Title/Abstract]) OR "cyclohexatriene"[Title/Abstract])) AND ((humans[Filter]) AND (english[Filter] OR french[Filter] OR german[Filter] OR italian[Filter] OR spanish[Filter]))
Embase (Ovid)	("benzene" or "'benzol" or "cyclohexa-1,3,5-triene" or "1,3,5-cyclohexatriene" or "cyclohexatriene").tw. and ("neoplasms" or "carcinoma" or "cancer" or "malignant").tw. limit to ((behavioral & social sciences or clinical medicine or health professions or life sciences or medical humanities or nursing or patient education or public health or science) and original articles)
Scopus	((TITLE-ABS-KEY (benzene) OR TITLE-ABS-KEY (benzol) OR TITLE-ABS-KEY (cyclohexa-1,3,5-triene) OR TITLE-ABS-KEY (1,3,5-cyclohexatriene) OR TITLE-ABS-KEY (cyclohexatriene))) AND ((TITLE-ABS-KEY (neoplasms) OR TITLE-ABS-KEY (carcinoma) OR TITLE-ABS-KEY (cancer) OR TITLE-ABS-KEY (malignant))) AND (LIMIT-TO (DOCTYPE , "ar") OR LIMIT-TO (DOCTYPE , "re")) AND (LIMIT-TO (SUBJAREA , "MEDI") OR LIMIT-TO (SUBJAREA , "ENVI")) AND (LIMIT-TO (LANGUAGE , "English") OR LIMIT-TO (LANGUAGE , "German") OR LIMIT-TO (LANGUAGE , "Italian") OR LIMIT-TO (LANGUAGE , "French") OR LIMIT-TO (LANGUAGE , "Spanish")) AND (LIMIT-TO (SRCTYPE , "j")) AND (EXCLUDE (SUBJAREA , "BIOC") OR EXCLUDE (SUBJAREA , "EART") OR EXCLUDE (SUBJAREA , "ENGI") OR EXCLUDE (SUBJAREA , "CENG")) AND (EXCLUDE (SUBJAREA , "COMP") OR EXCLUDE (SUBJAREA , "MATH")) AND (EXCLUDE (LANGUAGE , "Portuguese") OR EXCLUDE (LANGUAGE , "Turkish"))

Supplementary Table 3

MODIFIED NEWCASTLE - OTTAWA QUALITY ASSESSMENT SCALE

CASE CONTROL STUDIES (maximum score: 9)

Selection

1) Is the case definition adequate?

a) yes, with independent validation **(1)**
b) yes, eg record linkage **(1)** or based on self-reports **(0.5)**
c) no description **(0)**

2) Representativeness of the cases

a) consecutive or obviously representative series of cases **(1)**
b) potential for selection biases or not stated **(0)**

3) Selection of Controls

a) community controls **(1)**
b) hospital controls **(0.5)**
c) no description **(0)**

4) Definition of Controls

a) no history of disease (endpoint) **(1)**
b) no description of source **(0)**

Comparability

1) Comparability of cases and controls on the basis of the design or analysis

a) study controls for age, gender, province **(0)**
b) study controls for age, gender, province +smoking **(1)**
c) study controls for age, gender, province +smoking + other additional factors **(2)**

Exposure

1) Ascertainment of exposure

a) secure record (eg surgical records) **(1)**
b) structured interview where blind to case/control status **(1)**
c) interview not blinded to case/control status **(0.5)**
d) written self-report or medical record only **(0.5)**
e) no description **(0)**

2) Same method of ascertainment for cases and controls

a) yes **(1)**
b) no **(0)**

3) Non-Response rate

a) one or both groups over 90% **(1)**
b) one or both groups between 60- 90% **(0.5)**
c) one or both groups under 60% **(0)**
d) no statemen **(0)**

COHORT STUDIES (maximum score: 10)

Selection

1) Representativeness of the exposed cohort

a) truly representative of the average _______________ (describe) in the community **(2)**

b) somewhat representative of the average ______________ in the community **(1)**

c) selected group of users eg nurses, volunteers **(0.5)**

d) no description of the derivation of the cohort **(0)**

2) Selection of the non-exposed cohort

a) drawn from the same community as the exposed cohort **(1)**

b) drawn from a different source **(0.5)**

c) no description of the derivation of the non-exposed cohort **(0)**

3) Ascertainment of exposure

a) secure record (eg surgical records) **(1)**

b) structured interview **(1)**

c) written self-report **(0.5)**

d) no description **(0)**

4) Demonstration that outcome of interest was not present at start of study

a) yes **(1)**

b) no **(0)**

Comparability

1) Comparability of cohorts on the basis of the design or analysis

a) study controls for age, gender, province **(0)**

b) study controls for age, gender, province +smoking **(1)**

c) study controls for age, gender, province +smoking + other additional factors **(2)**

Outcome

1) Assessment of outcome

a) independent blind assessment **(1)**

b) record linkage **(1)**

c) self-report **(0.5)**

d) no description **(0)**

2) Was follow-up long enough for outcomes to occur

a) yes (select an adequate follow up period for outcome of interest) **(1) (average 15 years)**

b) no **(0)**

3) Adequacy of follow up of cohorts

a) complete follow up - all subjects accounted for over 90% **(1)**

b) subjects lost to follow up unlikely to introduce bias - small number lost - > ____ % (select an adequate %) follow up, or description provided of those lost) between 60- 90% **(0.5)**

c) follow up rate < ____% (select an adequate %) and no description of those lost under 60% **(0)**

d) no statemen **(0)**

Supplementary Table 4. Results on dose-response relationship

Cancer type	First Author, year	Exposure level	Dose detail	RR (95% CI)
Kidney	Gérin M (1998)	Low	N/A	1.2(0.7-1.9)
		Medium/High	N/A	1.3(0.7-2.4)
	Pesch B.(2000)	Medium	N/A	1.26(1.05-1.51)
		High	N/A	1.24(1.0-1.54)
	Vlaanderen, Jelle (2012)	Low (First T)	N/A	1(0.94-1.06)
		Medium (2T)	N/A	1(0.95-1.96)
		High (3T)	N/A	1.06(1-1.12)
	Wong O (1987)	Medium	25_100 ppm month	0.83 (0.06-5.94)
		High	>100 ppm month	1.54 (0.15-1.59)
Bladder	Hadkhale K (2017)	Low	<5.68ppm	1(0.92-1.08)
		Medium	5.68_15.04ppm	1.05(1-1.15)
		High	>15.04ppm	1.16(1.04-1.31)
	Wong O (1987)	Low	<25 ppm	3.89 (0.59-6.33)
		Medium	>100 ppm	0.55(0.09-8.96)
	Gérin M (1998)	Low	N/A	1(0.7-1.3)
		Medium	N/A	1.2(0.7-2)
		High	N/A	0.2(0-0.6)
	Shala NK (2023)	Low	>0-2	1.27(0.81-2.01)
		Medium	2-<7.6	1.11(0.69-1.77)
		High	7.6-<15.3	1.56(0.91-2.67)
		High	15.3-51.4	1.82(1.01-3.29)
	Xie Sh (2024)	Low (Q1)	N/A	1.72(0.99-2.99)
		Medium (Q2)	N/A	1.44(0.83-2.51)
		High (Q3)	N/A	1.99(1.15-3.43)
		High (Q4)	N/A	1.4(0.79-2.48)

Supplementary Table 5. Selected characteristics of the studies included in the review and meta-analysis.

First author name, years of publication	Country	Gender	Type Of Study	Industry	Cancer type	Outcome	Variables adjusted for in the analysis other than gender, age and calendar period	Quality assessment score
Rushton L,1980	UK	Men	Cohort	Oil Industry	Bladder	Mortality	region	7
Guberan E, 1985	Switzerland	Men	Cohort	Perfumery and Flavour Industry	Kidney, Bladder, And Other Urinary Organs	Incidence, Mortality	-	8
Bond GG, 1986	USA	Both	Cohort	Mixed	Kidney, Bladder	Mortality	-	7
Wong O, 1987	USA	Male	Cohort	Chemical industry	Kidney, Bladder	Mortality	-	8
Wongsrichanalai C., 1989	USA	Men	Cohort	Petroleum Refinery	Kidney, Bladder	Incidence	-	8
Steineck G,1990	Sweden	Men	Case Control		Urothelial Cancer	Incidence	Year of birth and smoking	7
Szeszenia-Dabrowska N, 1991	Poland	Men	Cohort	Rubber Workers	Kidney, Bladder, And Other Urinary Organs	Mortality	-	6
Dolin PJ, 1992	UK	Both	cohort	Benzene Exposure Job	Bladder	Mortality	-	7
Walker JT, 1993	USA	Both	Cohort	Shoe Manufactoring	Kidney, Bladder, And Other Urinary Organs	Mortality	-	9
Lagorio S, 1994	Italy	Men	Cohort	Service Station	Kidney, Bladder	Mortality	-	7
Greenland S, 1994	USA	Men	Case Control	Transformer-Assembly Facility	Kidney, Bladder	Mortality	Hire year, death year	6
Honda Y, 1995	USA	Men	cohort	Petroleum Manufacturing Plant	Kidney, Bladder	Mortality	-	8
Satin K.P., 1996	USA	Both	Cohort	Oil Refinery	Bladder And Other Urinary Organs	Mortality	-	7
Collingwood KW, 1996	USA	Both	Cohort	Mixed	Kidney, Bladder	Mortality	-	8
Fu H, 1996	UK	Men	English Cohort/ Italy cohort	Shoe Manufactoring	Kidney, Bladder	Mortality	-	8
Lynge E, 1997	European Countries	Both	Cohort	Service Station	Bladder And Other Urinary Organs	Incidence	-	7
Järvholm B, 1997	Swedes	Men	Cohort	Oil Refinery	Kidney And Urinary Bladder	Incidence	-	8
Pukkala, E, 1998	Finland	Both	Cohort	Oil Refinery	Kidney, Bladder	Incidence	-	7
Gérin M, 1998	Montreal	Both	Case Control		Kidney, Bladder	Incidence	family income, ethnic group, cigarette smoking, respondent status, exposure to aromatic amines	8.5
Consonni D., 1999	Italy	Men	Cohort	Oil Refinery	Bladder, Urinary Track	Mortality	-	8
Bulbulyan MA, 1999	Russia	Women	Cohort	Printing Industry	Kidney, Bladder	Mortality	-	8
Pesch B., 2000	Germany	Both	Case Control	Mineral Oils and Related Products	Renal Cell	Incidence	study center, and smoking	7.5
Hu J, 2002	Canada	Both	Case Control	Chemical Industry	Renal Cell	Incidence	Adjusted for 10-year age groups, province, education, BMI (<20, 20–27, >27), pack-years of smoking, alcohol use and total consumption of meat	8

Kauppinen T, 2003	Finland	Both	Finnish Cohort	Chemical Laboratory	Kidney, Bladder, And Other Urinary Organs	Incidence	-	8
Lewis, R J, 2003	Canada	Both	Cohort	Petroleum Workers	Kidney, Bladder	Incidence, Mortality	-	8
Sorahan T, 2005	UK	Both	Cohort	Benzene Exposed Workers	Kidney, Bladder, And Other Urinary Organs	Incidence, Mortality	-	8
Gun RT, 2006	Australia	Men	Cohort	Petroleum Industry	Kidney, Bladder	Incidence, Mortality	-	8.5
Hoshuyama T., 2006	China	Men	Cohort	Iron and Steel Workers	Bladder	Mortality	-	8
Budroni M, 2010	Italy	Both	Cohort	Petrochemical industry	Kidney, Bladder	Incidence	-	6
Koh DH,2011	South Korea	Male	cohort	Petrochemical industry	Bladder	Incidence	-	7.5
Bonneterre V, 2012	France	Both	Cohort	Chlorochemical Plant	Kidney, Bladder	Incidence	-	7.5
Vlaanderen, Jelle, 2012	Nordic Countries	Both	Cohort	Benzene Exposed Job	Kidney	Incidence	-	6
Anttila A., 2015	Finland	Both	Nested Case Control	Oil Refining	Kidney	Incidence	year of birth (i.e., age) and sex	6
Ott MG, 2015	USA	Men	Cohort	Occupational Exposed to Benzene	Urinary Organs	Mortality	-	8
Hadkhale K, 2017	Nordic Countries	Both	Case Control	Benzene Exposed Jobs	Bladder	Incidence	adjusted for exposure to other solvents and chemicals.	6
Collins J, 2015	USA	Men	cohort	Benzene Exposed Jobs	Kidney, Bladder	Mortality	-	8
Linet M,2015	China	Both	Cohort	Multiple industries	Bladder	Mortality	-	8
Gustavsson P, 2017	Swedes	Both	Cohort	Chemical Laboratory	Kidney, Bladder, And Other Urinary Organs	Incidence	-	8
Shala NK, 2023	Norway	Male	Cohort	Petroleum	Bladder	Incidence	Adjusted for year of first employment, tobacco smoking, education and a summary PAH-proxy variable.	8
Xie Sh, 2024	USA	Both	Case control	Benzene Exposed Jobs	Bladder	Incidence	Adjusted for smoking status, state, race, ethnicity (Hispanic) and non-solvent exposed high-risk occupations for bladder cancer.	8
